# Combined Antineoplastic Effects of Metformin, Boric Acid and Resveratrol in SKOV3 Ovarian Cancer Cells

**DOI:** 10.3390/biomedicines14030719

**Published:** 2026-03-20

**Authors:** Burcu Biltekin, Mete Hakan Karalok, Seyma Dumur, Naile Fevziye Mısırlıoglu, Hafize Uzun

**Affiliations:** 1 Department of Histology and Embryology, Faculty of Medicine, Istanbul Atlas University, Istanbul 34408, Turkey; 2 Department of Obstetrics and Gynecology, Faculty of Medicine, Istanbul Atlas University, Istanbul 34408, Turkey; hakan.karalok@atlas.edu.tr; 3 Department of Biochemistry, Faculty of Medicine, Istanbul Atlas University, Istanbul 34408, Turkey; seyma.dumur@atlas.edu.tr (S.D.); nailemisirlioglu@gmail.com (N.F.M.); huzun59@hotmail.com (H.U.)

**Keywords:** ovarian cancer, SKOV3 cells, metformin, boric acid, resveratrol, IL-17, NF-κB, midkine

## Abstract

**Background:** Ovarian cancer (OC) is characterized by aggressive progression, high metastatic potential, and frequent resistance to conventional chemotherapy, highlighting the need for novel combination-based therapeutic strategies. Metformin has emerged as a promising antineoplastic agent; however, its efficacy may be enhanced through combination with bioactive compounds. This study aimed to investigate the antineoplastic effects of metformin in SKOV3 human OC cells and to evaluate whether these effects could be potentiated by boric acid (BA) and resveratrol, with particular emphasis on their modulatory impact on key inflammatory and tumor-associated biomarkers, including interleukin-17 (IL-17), nuclear factor kappa-B (NF-κB), and midkine (MDK). **Methods:** SKOV3 cells were treated with metformin, BA, and resveratrol as monotherapies or in combination. Cell viability was assessed using a colorimetric assay, while migratory capacity was evaluated by wound healing analysis. The expression levels of IL-17, NF-κB, and MDK were quantified in cell lysates, and p21 protein expression was analyzed by immunocytochemistry. **Results:** All treatments induced concentration- and time-dependent reductions in cell viability. Combination treatments, particularly metformin with boric acid or resveratrol, produced more pronounced inhibitory effects on cell survival and migration compared with single-agent treatments. Inflammatory and tumor-associated biomarkers, including IL-17, NF-κB, and MDK, were significantly modulated following treatment. Additionally, increased p21 expression was observed in treated cells, indicating enhanced cell cycle regulatory activity. **Conclusions:** These findings indicate that BA and resveratrol enhance the antineoplastic activity of metformin in SKOV3 OC cells by suppressing proliferative and migratory capacities and modulating inflammatory mediators such as IL-17, NF-κB, and MDK. However, since toxicity assessments in non-cancerous cells were not performed, the safety profile of this combination remains unclear and requires further investigation in non-cancerous models.

## 1. Introduction

Ovarian cancer (OC) has the highest mortality among gynecological malignancies, with more than 300,000 new cases diagnosed worldwide each year and a 5-year survival rate below 50% [1]. Delayed diagnosis due to nonspecific early symptoms, along with a strong tendency for peritoneal dissemination, ascites formation, and the development of chemotherapy resistance, remains a major clinical challenge [2]. Accumulating epidemiological evidence highlights chronic inflammation as a key contributor to OC development, as reflected by the markedly increased cancer risk observed in women with endometriosis or a history of pelvic inflammatory disease [3]. Despite advances in surgical debulking and systemic therapy, overall survival rates have shown only modest improvement, highlighting the need for novel and more effective therapeutic strategies [4].

Metformin, a biguanide widely prescribed for type 2 diabetes mellitus, has attracted growing interest for its antineoplastic properties. Preclinical and clinical evidence indicates that metformin inhibits cancer cell proliferation, migration, and survival through modulation of cellular metabolism, AMP-activated protein kinase (AMPK) signaling, and cell cycle regulators such as p21 [5,6]. However, the anticancer efficacy of metformin appears to be dose- and context-dependent, suggesting that combination-based approaches may be required to maximize its therapeutic potential [6,7].

Boron (B) is a naturally occurring trace element that exists predominantly as boric acid (BA) in biological systems. Owing to its efficient absorption and systemic distribution, BA exhibits antioxidant and immunomodulatory properties. Growing evidence indicates that boron-containing compounds possess anticarcinogenic activity across multiple malignancies, including ovarian, prostate, cervical, lung, breast, and melanoma, and may serve as a natural adjunct to reduce treatment-related adverse effects in OC [8,9,10,11,12,13].

Resveratrol (3,4′,5-trihydroxy-trans-stilbene) is a naturally occurring polyphenolic phytoalexin predominantly found in grapes, peanuts, and blueberries [14]. Substantial experimental evidence indicates that resveratrol exerts anticarcinogenic effects in both hematological and solid tumors, including breast, liver, colorectal, pancreatic, and prostate cancers [15,16]. These effects are mediated through the modulation of multiple cancer-related signaling pathways, such as PI3K/AKT/mTOR, p53, MAPKs, NF-κB, and STAT3, in a context-dependent manner [17].

Chronic inflammation plays a critical role in OC progression and metastasis [18]. Proinflammatory mediators such as interleukin-17 (IL-17) and nuclear factor kappa-B (NF-κB) contribute to tumor growth, cell survival, and migratory capacity, while MDK, a heparin-binding growth factor, is increasingly recognized as a tumor-associated biomarker involved in proliferation, angiogenesis, and treatment resistance [18,19,20,21,22]. Targeting these interconnected signaling pathways may therefore represent an effective approach to improving therapeutic outcomes in OC.

The present study aimed to investigate the antineoplastic effects of metformin in the SKOV3 human OC cell line and to evaluate whether these effects could be enhanced through combination with boric acid and resveratrol. Specifically, the study assessed the impact of these treatments on cell viability, migratory behavior, and cell cycle regulation, as well as their modulatory effects on key inflammatory and tumor-associated biomarkers, including IL-17, NF-κB, and MDK. By exploring the combined effects of these agents, this study sought to provide preliminary mechanistic insights into potential combination-based therapeutic strategies for OC.

## 2. Materials and Methods

### 2.1. SKOV3 Cell Culture

SKOV3 human OC cells from ATCC (Manassas, VA, USA) were maintained in RPMI-1640 medium (Gibco, Thermo Fisher Scientific, Waltham, MA, USA) supplemented with 10% FBS (Sigma-Aldrich, St. Louis, MO, USA) and 1% penicillin–streptomycin (Sigma-Aldrich, St. Louis, MO, USA) at 37 °C in a 5% CO_2_ atmosphere. To ensure exponential growth, cells were passaged twice weekly and harvested at 80% confluence. Metformin and BA stock solutions were prepared in complete medium and diluted to working concentrations immediately before use. For all functional assays—including viability, migration, and immunocytochemistry—cells were treated for 24–72 h. Control groups were maintained under identical conditions without pharmacological intervention. All experiments were performed in triplicate to ensure reproducibility. SKOV3 cells were selected as the experimental model because they represent a well-established ovarian cancer cell line widely used in experimental oncology studies. This cell line is characterized by high proliferative and migratory capacity and a p53-null background, making it suitable for evaluating antiproliferative and antimigratory responses to therapeutic agents. However, considering the molecular and phenotypic heterogeneity of ovarian cancer, the use of a single cell line represents a limitation, and future studies including additional ovarian cancer cell lines will be important to validate the broader applicability of these findings.

**Figure 1:**
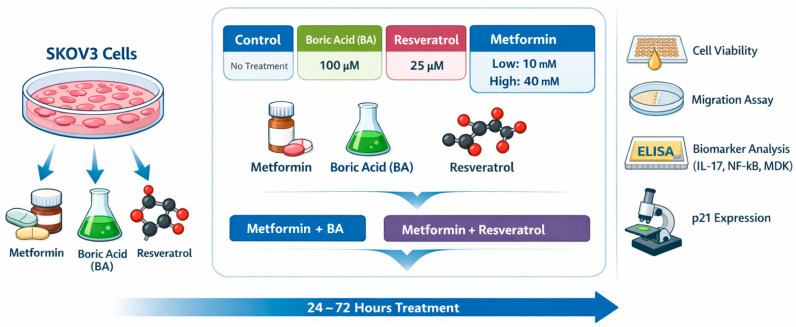
Experimental design and treatment workflow for SKOV3 ovarian cancer cells. SKOV3 cells were treated with metformin, boric acid (BA), and resveratrol as monotherapies or in combination for 24–72 h. Following treatment, cell viability was evaluated using the CCK-8 assay, migratory capacity was assessed by wound healing assay, inflammatory and tumor-associated biomarkers (IL-17, NF-κB, and MDK) were quantified, and p21 protein expression was analyzed by immunocytochemistry.

Schematic overview of the experimental workflow and treatment conditions applied to the SKOV3 cells were summarized in Figure 1 and Table 1. SKOV3 cells were treated with metformin, BA, and resveratrol as monotherapies or in combination and evaluated sequentially for cell viability, migratory capacity, biomarker modulation, and cell cycle regulation all of which are explained in the following sections.

### 2.2. Cell Viability Assays

SKOV3 cells were seeded into 96-well plates at a density of 1.5 × 10^4^ cells/well and allowed to adhere for 24 h. Based on literature review, the cells were then treated with varying concentrations of metformin (10–50 mM), BA (10–100 μM), and resveratrol (25–100 μM) for up to 72 h. Cell viability was assessed using a colorimetric CCK-8 assay (Abbkine, Wuhan, China) per the manufacturer’s instructions. After adding 10 μL of CCK-8 reagent to each well, the plates were incubated for 3 h at 37 °C. The optical density (OD) was measured at 450 nm utilizing a microplate reader (Bio-Tek, Winooski, VT, USA). All experiments were conducted in triplicate, and viability was expressed relative to the untreated control group.

### 2.3. Wound Healing Assay

SKOV3 cells were seeded in 6-well plates and grown to 80% confluence. A standardized scratch was made in the monolayer using a sterile 100 µL pipette tip held at a constant perpendicular angle to ensure a uniform initial wound width across all wells. Debris was removed by washing twice with 1× PBS. The cells were then treated with Metformin, Boric acid, and Resveratrol at specified concentrations. Migration distance was quantified by measuring the wound gap width in micrometers (µm) from captured images using the Micro-Image Analysis System software (V1.1.6, MShot.Main, Micro-Image Analysis System). For each experimental group, three representative fields were analyzed, and the mean value of these measurements was used to calculate migration. To quantify the extent of healing, the mean distance between wound edges was measured at multiple points and normalized to the 0 h baseline. In addition, quantitative migration inhibition percentages were calculated relative to the control group using the formula: Migration inhibition (%) = [1 − (wound closure of treated group/wound closure of control group)] × 100.

### 2.4. Immunocytochemical Analysis of p21

SKOV3 cells were seeded onto coverslips and cultured for 24 h before being exposed to Metformin, Boric Acid, and Resveratrol at designated concentrations and durations. All assays were performed in triplicate to ensure experimental reproducibility. Following treatment, cells were fixed in chilled methanol (−20 °C) for 5 min.

To evaluate the antiproliferative potential and cell cycle regulation, protein expression of p21 was determined using the indirect streptavidin–biotin immunoperoxidase method. Briefly, coverslips were incubated overnight at 4 °C with a primary antibody against p21 (Affinity, AF6290; 1:100 dilution) according to the manufacturer’s protocol. Immunoreactivity was detected using an anti-polyvalent HRP kit (SensiTek, ScyTek Laboratories, Logan, UT, USA) and visualized with the AEC Substrate Detection System (ScyTek).

The intensity of p21 expression was quantified semi-quantitatively via a modified H-SCORE analysis assigning numerical scores between 0 and 300 to the immunostaining, a method used by Yaprak et al. [23]. This scoring system integrated both the staining intensity and the percentage of positively stained cells to provide a comprehensive assessment of immunoreactivity. For each sample, five representative high-power fields were evaluated, and the percentage of positive cells was determined across these fields to calculate the final H-score.

### 2.5. Measurement of Inflammatory Marker

Samples (lysate) were processed under standardized conditions and were stored at −80 °C until analysis.

#### 2.5.1. Assessment of Human Midkine (MDK)

MDK concentrations were quantified using a commercially available Human Midkine (MDK) ELISA kit (cat. no: KTE61678; Abbkine Scientific Co., Ltd., Wuhan, China), in accordance with the manufacturer’s instructions. All samples were thawed once and analyzed simultaneously to minimize inter-assay variability. Standards and samples were added to pre-coated microplate wells and incubated to allow binding of MDK to the immobilized antibodies. MDK concentrations were calculated from the standard calibration curve and expressed as ng/mL. The intra- and inter-assay coefficients of variation were <10%, as reported by the manufacturer.

#### 2.5.2. Assessment of Interleukin-17 (IL-17)

IL-17 levels were determined using a quantitative ELISA kit specific for human IL-17 (ELK Biotechnology Co., Ltd., Wuhan, China), following the manufacturer’s protocol. IL-17 concentrations were calculated using a standard curve generated by known concentrations of recombinant IL-17 and expressed as pg/mL.

#### 2.5.3. Assessment of Nuclear Factor Kappa-B (NF-κB)

NF-κB levels were measured using a human NF-κB ELISA kit (ELK Biotechnology Co., Ltd., Wuhan, China) according to the manufacturer’s instructions. Briefly, samples and standards were incubated in antibody-coated microplate wells to allow binding of NF-κB. NF-κB concentrations were determined from a standard curve and expressed as ng/mL.

All samples were analyzed intra and inter-assay variability remained below 10%. ELISA values were normalized to the untreated control group and expressed relative to control levels to facilitate comparison between experimental conditions and minimize inter-assay variability. ELISA measurements were conducted in technical duplicates to ensure measurement reliability.

All experiments were performed using three independent biological replicates to ensure reproducibility and reliability of the results.

### 2.6. Statistical Analysis

Statistical analyses were performed using GraphPad Prism version 8.0.1 (GraphPad Software, San Diego, CA, USA). Normality of the data distribution was evaluated using the Kolmogorov–Smirnov test prior to statistical analysis. Since the data did not meet the assumptions of normality, non-parametric tests were applied. Comparisons among multiple independent groups were performed using the Kruskal–Wallis test. When a statistically significant difference was detected, Dunn’s multiple comparisons post hoc test was used to identify differences between individual groups. Results were expressed as mean ± standard deviations where appropriate. A *p* value of <0.05 was considered statistically significant.

## 3. Results

Initial viability assessments demonstrated time- and dose-dependent reductions in cell survival, which were more pronounced in combination treatments. Wound healing assays revealed significant inhibition of cell migration over 72 h. Treatment further resulted in marked modulation of inflammatory and tumor-associated biomarkers, including decreased IL-17 and NF-κB levels and altered MDK expressions. Immunocytochemical analysis showed increased p21 expression in treated cells, indicating enhanced cell cycle arrest.

### 3.1. Effects of Metformin, Boric Acid, and Resveratrol on SKOV3 Cell Viability

**Figure 2:**
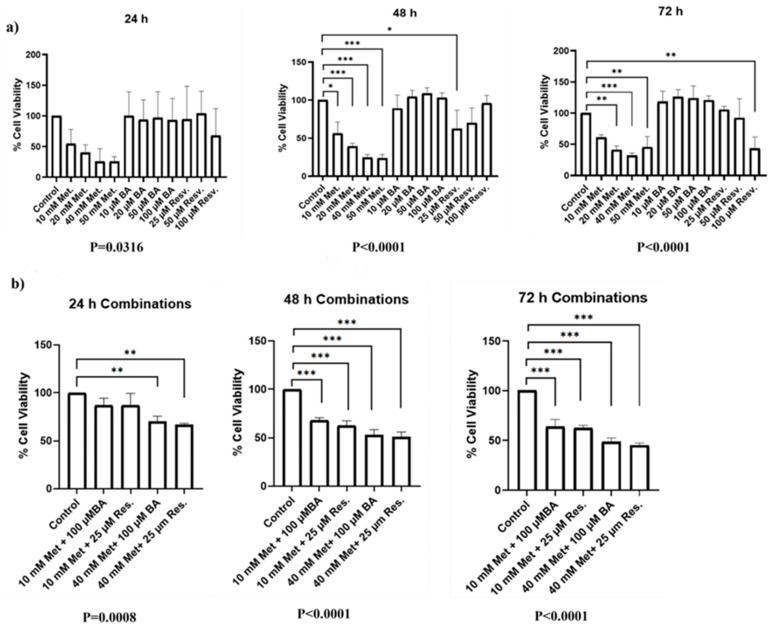
Dose–response characterization and temporal viability profiles of SKOV3 cells. This figure illustrates the cytotoxic effects of metformin, boric acid, and resveratrol on the SKOV3 ovarian cancer cell line. Cell viability was monitored over a 24–72 h period using the CCK-8 assay. (**a**) Dose–response curves for each agent administered as monotherapy, demonstrating a concentration-dependent reduction in cell viability. (**b**) Effects of combination treatments compared with single-agent exposure across the indicated time points. Data are presented as the mean percentage of viable cells relative to untreated controls. * *p* < 0.05; ** *p* < 0.01; *** *p* < 0.001, all of them are vs. control.

The cytotoxic effects of metformin, boric acid (BA), and resveratrol on SKOV3 cells were evaluated using the CCK-8 assay over a 24–72 h period. As shown in Figure 2a, all three agents exerted concentration- and time-dependent reductions in cell viability when administered as monotherapies. Higher concentrations and longer exposure times were associated with a more pronounced decrease in viable cell percentages compared with untreated controls. At 24 h, the estimated half-maximal inhibitory concentration (IC_50_) value for metformin was approximately 16.3 mM, whereas the IC_50_ values for BA and resveratrol exceeded 100 µM under the tested conditions. At 48 h, the estimated IC_50_ value for metformin was approximately 13.7 mM, whereas the IC_50_ values for BA and resveratrol remained above 100 µM within the tested concentration range. At 72 h, the estimated IC_50_ values were approximately 15.5 mM for metformin and 93.2 µM for resveratrol, whereas the IC_50_ value for boric acid remained above 100 µM within the tested concentration range.

Combination treatments further modulate cell viability (Figure 2b). Several metformin-based combinations with BA or resveratrol resulted in greater reductions in cell survival compared with the corresponding single-agent treatments. These findings suggest greater reductions in cell viability in the combination groups compared with the corresponding single-agent treatments. Cell viability data are expressed as percentages relative to untreated control values.

### 3.2. Inhibitory Effects on SKOV3 Cell Migration

**Figure 3:**
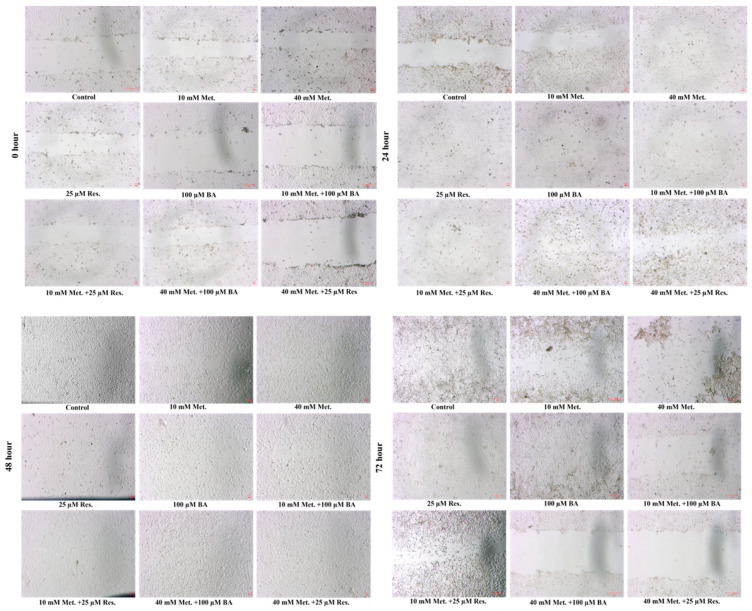
Wound healing images of SKOV3 cells. ×40 magnifications.

The migratory capacity of SKOV3 cells was evaluated using the wound healing assay. Representative images obtained at 0, 24, 48, and 72 h showed progressive wound closure in the control group, whereas treatment with metformin, BA, and resveratrol significantly delayed this process (Figure 3). Treated cells exhibited reduced motility and slower wound closure compared with untreated controls, indicating an inhibitory effect of the treatments on SKOV3 cell migration.

Quantitative analysis of migration inhibition percentages derived from wound closure measurements is summarized in Table 2. BA and resveratrol monotherapies showed modest inhibitory effects on migration, although these changes were not consistently statistically significant across time points. Metformin monotherapy produced variable inhibition levels, with higher inhibition percentages observed particularly at later time points. Notably, several combination treatments demonstrated stronger inhibitory effects on cell migration. In particular, 40 mM metformin combined with 100 µM BA and 40 mM metformin combined with 25 µM resveratrol produced the highest migration inhibition percentages, reaching approximately 92.7% and 67.9% at 72 h, respectively. Overall, metformin-based combinations tended to show greater inhibition of migration compared with single-agent treatments.

### 3.3. Modulation of Inflammatory and Tumor-Associated Biomarkers

**Figure 4:**
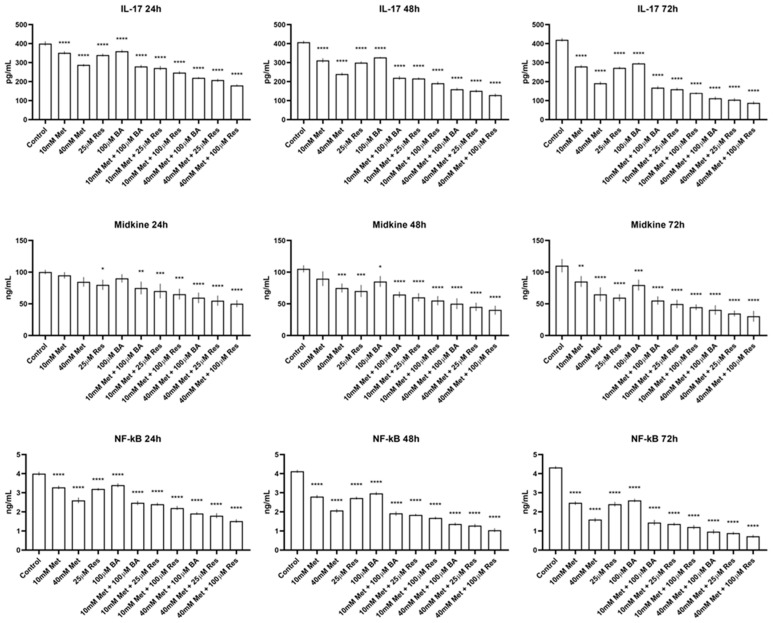
Impact of treatments on IL-17, MDK, and NF-κB protein levels in SKOV3 cells. IL-17 and NF-κB levels were reduced following treatment with metformin, BA, and resveratrol, while MDK levels also decreased in response to metformin-based treatments. Data are presented as mean ± SD of three independent experiments. Statistical significance between control and treated groups is indicated by asterisks (*). * *p* < 0.05; ** *p* < 0.01; *** *p* < 0.001, **** *p* < 0.0001, all of them are vs. control.

The effects of metformin, BA, and resveratrol on IL-17, MDK, and NF-κB protein levels were evaluated in SKOV3 cell lysates. As shown in Figure 4, treatment with metformin, BA, and resveratrol significantly altered the levels of all three biomarkers compared with control cells. IL-17 and NF-κB levels were consistently reduced following treatment, with more pronounced decreases observed in the combination groups. MDK levels also decreased across most treatment groups. Metformin treatment reduced MDK levels by approximately 10–20% at 24 h and 20–30% at later time points, while resveratrol treatment produced reductions of approximately 25–40%. BA treatment resulted in more moderate decreases of approximately 10–25% depending on the exposure duration. Combination treatments produced the greatest reductions, with MDK levels decreasing by approximately 35–60% compared with control values, particularly at 48 h and 72 h. These findings suggest that the applied treatments influence key inflammatory and tumor-associated signaling molecules. Data are presented as mean ± SD of three independent experiments, and statistically significant differences relative to controls are indicated.

### 3.4. Effects of Treatments on p21 Protein Expression

**Figure 5:**
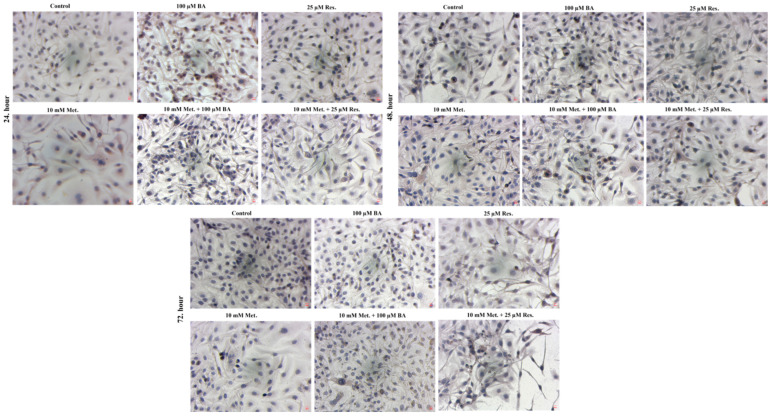
Immunohistochemical analysis of p21 expression in SKOV3 cells. Images were captured at ×200 magnification.

Immunocytochemical analysis demonstrated that treatment with metformin, BA, and resveratrol increased p21 expression in SKOV3 cells. Representative micrographs (Figure 5) revealed enhanced p21-positive nuclear staining in treated groups compared with controls, indicating activation of cell cycle regulatory mechanisms.

Semi-quantitative evaluation using the H-SCORE method demonstrated significantly higher p21 immunoreactivity in several treatment groups compared with control cells (Table 3). At 24 h, metformin treatment resulted in higher p21 levels, and combination treatments further increased p21 expression, particularly in the metformin + BA group. At 48 h, p21 expression continued to increase, with the highest levels observed in the metformin + BA and metformin + resveratrol groups. By 72 h, the most pronounced upregulation of p21 was detected in metformin-containing treatments, including metformin alone (median 68.18), metformin + BA (median 68.75), and metformin + resveratrol (median 70.43), all of which were significantly higher than control levels. These findings indicate a time-dependent increase in p21 expression following treatment, particularly in metformin-based regimens, suggesting activation of cell cycle regulatory mechanisms.

## 4. Discussion

OC is the leading cause of death among gynecological malignancies worldwide. The most important finding of the present study is that metformin-based combination treatments can enhance antineoplastic responses in SKOV3 OC cells, resulting in coordinated suppression of cell viability and migratory capacity together with modulation of key inflammatory and tumor-associated pathways. Compared with monotherapies, several combination regimens—particularly those including higher concentrations of metformin with BA or resveratrol—demonstrated greater inhibition of cell migration, as reflected by increased migration inhibition percentages. These treatments were also associated with significant alterations in IL-17, NF-κB, and MDK levels, suggesting attenuation of inflammation-related tumor signaling. In addition, the observed upregulation of p21 indicates reinforcement of cell cycle regulatory mechanisms. Collectively, these findings support a multifaceted mode of action in which metformin-based combination strategies may influence both proliferative and inflammatory processes relevant to OC progression. IL-17, NF-κB, and MDK were evaluated as representative inflammatory and tumor-associated biomarkers involved in ovarian cancer progression and tumor microenvironment signaling. Previous studies indicate that metformin and resveratrol can influence inflammatory pathways, particularly those related to NF-κB signaling. Accordingly, these biomarkers were analyzed to explore potential inflammation-related responses to the treatments; however, the present study does not establish a direct mechanistic link between these pathways and the observed cellular effects.

### 4.1. Effects of Metformin, BA, and Resveratrol on SKOV3 Cell Viability

In this study, metformin, BA, and resveratrol reduced SKOV3 cell viability in a time- and dose-dependent manner. Moreover, metformin-based combination treatments resulted in greater reductions in cell viability compared with single-agent exposure across the examined time points. These findings align with a substantial body of evidence showing that metformin suppresses proliferation across multiple OC models, including SKOV3-related lines, frequently accompanied by cell-cycle arrest with increased p21 and suppression of mTOR signaling via AMPK-associated metabolic stress [7]. In the present study, metformin reduced SKOV3 cell viability in a time- and dose-dependent manner, and this inhibitory effect was further enhanced when metformin was combined with BA or resveratrol. These observations agree with previous OC studies demonstrating that metformin suppresses tumor cell growth and proliferation [24,25,26,27,28,29,30]. The more pronounced reduction in viability observed over the 24–72 h treatment period in the combination groups supports the concept that metformin-based therapeutic strategies may achieve greater antitumor efficacy when used alongside complementary bioactive compounds [26,27,30]. It should be noted that some of the metformin concentrations used in the present in vitro experiments exceed clinically achievable plasma levels. Although such concentrations are commonly employed in preclinical studies to investigate cellular and mechanistic responses, the translational relevance of these findings should be interpreted with caution. Future studies using pharmacologically relevant concentrations and more physiologically representative models will be important to better evaluate the clinical applicability of the observed effects.

Resveratrol has likewise been repeatedly shown to decrease viability and promote apoptosis-related signaling in SKOV3 contexts through multi-pathway modulation (e.g., stress-activated kinases and inflammatory transcriptional programs), and recent work supports suppression of pro-tumor signaling linked to NF-κB in OC models [31]. Therefore, the greater decline in viability in combination groups is biologically plausible, as metformin-driven metabolic restriction can sensitize tumor cells to additional stressors, while resveratrol can concurrently dampen survival signaling and reinforce cytotoxic stress responses [32,33].

For BA, growing experimental literature indicates dose-dependent antiproliferative activity of boric acid/boron-containing compounds across cancer types, with proposed mechanisms that include altered signaling and stress responses and, in some models, apoptosis-related programs [34]. Although OC-specific data on BA remain limited, the present findings demonstrate that BA exerts measurable growth-inhibitory effects in SKOV3 cells and enhances the antiproliferative activity of metformin and resveratrol when used in combination, supporting a contributory role for BA within metformin-based treatment strategies [35].

From a translational standpoint, these viability findings strengthen the rationale for metformin-centered combination strategies in OC, an area already supported by mechanistic and preclinical evidence suggesting reduced tumor growth and pathway-level suppression relevant to progression and resistance [35]. Future work should quantify combination effects formally (e.g., Bliss/Loewe/ZIP synergy scores), validate across additional OC lines and 3D models, and benchmark exposures against clinically achievable ranges to improve clinical interpretability [36].

### 4.2. Inhibitory Effects on SKOV3 Cell Migration

Previous studies have demonstrated that metformin inhibits OC cell proliferation and invasion in both in vitro and in vivo models [37,38]; however, the underlying molecular mechanisms remain incompletely understood. The present study demonstrates that metformin, BA, and resveratrol influence the migratory behavior of SKOV3 OC cells, with several treatment groups—particularly metformin-containing combinations—producing greater inhibition of wound closure compared with certain monotherapies. Given that cell migration is a critical determinant of peritoneal dissemination and metastatic progression in OC, these findings are of relevance. Previous studies have shown that metformin can impair OC cell motility by altering cytoskeletal dynamics and energy metabolism, while resveratrol has been reported to suppress migration through modulation of signaling pathways involved in epithelial–mesenchymal transition and inflammatory regulation [26,27,37,38]. The enhanced antimigratory responses observed in some combination groups suggest that simultaneous targeting of metabolic stress and signaling pathways may contribute to restricting OC cell movement. Collectively, these data indicate that metformin-based combination strategies may interfere with key migratory processes that underline OC progression [39,40].

In the wound healing assay, treatment with metformin, BA, and resveratrol was associated with reduced wound closure in SKOV3 cells. However, because these agents also decreased cell viability and a proliferation inhibitor such as mitomycin C was not included in the assay design, the observed reduction in wound closure may partly reflect decreased cell proliferation in addition to impaired migratory capacity. Therefore, the relative contribution of proliferation and migration cannot be fully distinguished in the present study. Future studies incorporating proliferation inhibitors, shorter observation intervals, and complementary migration assays such as Transwell or Boyden chamber systems will be important to more precisely evaluate migration-specific effects.

### 4.3. Effects of Treatments on p21 Protein Expression

p21 was expressed in OC tissues without p53 expression [41]. Nuclear expression of p21 has been documented in various malignancies, including OC [42,43,44]. In the present study, treatment with metformin, BA, and resveratrol resulted in a marked increase in p21 protein expression in SKOV3 OC cells, with the most pronounced effect observed in the combination groups. p21 is a critical cyclin-dependent kinase inhibitor that regulates G1/S cell cycle progression and is frequently implicated in growth arrest and antiproliferative responses in cancer cells. Previous studies have demonstrated that metformin can induce p21 expression in ovarian and other cancer cell models, contributing to cell cycle arrest independently of p53 status and in association with metabolic stress signaling pathways [7,45,46]. Resveratrol has likewise been shown to upregulate p21 expression in OC cells, where it promotes growth inhibition and reinforces cell cycle checkpoint control through modulation of stress-responsive and inflammatory signaling cascades [47,48]. Although data regarding BA-mediated regulation of p21 in OC are limited, experimental evidence from other cancer models suggests that boron-containing compounds can influence cell cycle-related proteins, including p21, thereby contributing to antiproliferative effects [8,49,50]. The concurrent upregulation of p21 observed in this study supports the notion that metformin-based combination strategies may converge on cell cycle regulatory mechanisms, providing a complementary route to suppress OC cell proliferation. Inhibition of p21 was shown to induce cell death in cells exhibiting high p21 expression, indicating that targeting p21 may represent a potential therapeutic strategy for the treatment of OC [51]. Although increased p21 expression and modulation of IL-17, NF-κB, and MDK levels were observed following treatment, the present study does not establish a direct mechanistic relationship between these molecular changes. p21 upregulation was primarily interpreted as an indicator of cell cycle regulation associated with the growth-inhibitory effects of the treatments, whereas the alterations in inflammatory biomarkers may reflect broader cellular responses. Further mechanistic studies will be required to determine whether p21-mediated cell cycle regulation is functionally linked to these inflammatory signaling pathways.

### 4.4. Modulation of Inflammatory and Tumor-Associated Biomarkers

Chronic inflammation plays a central role in cancer development by contributing to multiple stages of tumor initiation, progression, and dissemination [52,53]. In the present study, treatment with metformin, BA, and resveratrol significantly modulated key inflammatory and tumor-associated biomarkers, including IL-17, NF-κB, and MDK, in SKOV3 OC cells. IL-17 is recognized as a pro-tumorigenic cytokine that promotes cancer cell survival, angiogenesis, and metastatic behavior, largely through activation of NF-κB-dependent inflammatory signaling. Previous studies suggest that metformin can influence inflammatory signaling pathways in the tumor microenvironment. For example, metformin-primed cancer-associated fibroblasts accompanied by reduced NF-κB/IL-6 activation may alter tumor survival signals and macrophage recruitment, thereby influencing OC progression [54]. In addition, metformin has been reported to suppress OC progression by downregulating TRIM37 and attenuating NF-κB pathway activation, resulting in decreased expression of invasion-associated proteins [55]. However, clinical evidence regarding the benefits of metformin in OC remains inconsistent, with some studies reporting no significant improvement in overall survival and emphasizing the need for more robust epidemiological data [56,57].

NF-κB is a central transcription factor linking chronic inflammation to OC progression and therapeutic resistance. Several pro-angiogenic and inflammatory mediators, including VEGF and IL-8, are transcriptionally regulated by NF-κB. Resveratrol, a polyphenolic antioxidant found in red grape skins, has demonstrated anti-inflammatory and anticancer properties and has been shown to inhibit NF-κB signaling and pro-angiogenic mediators in several malignancies, although its role in regulating NF-κB-related pathways in OC remains incompletely understood [58]. In parallel, MDK is an oncogenic growth factor produced by tumor, stromal, and immune cells and is implicated in cancer cell proliferation, invasion, and poor prognosis, highlighting the MDK axis as a potential therapeutic target in women’s cancers [59]. Within this context, modulation of MDK-associated signaling by metformin-based strategies may contribute to antitumor responses [60].

Consistent with these observations, the reduction in IL-17 levels observed in the present study was accompanied by suppression of NF-κB, suggesting attenuation of an inflammation-driven oncogenic axis. In addition, MDK levels were significantly modulated following treatment, further supporting the involvement of tumor-associated signaling pathways. Together, these findings suggest that metformin-based combination strategies may exert antineoplastic effects not only by suppressing cellular proliferation and migration but also by modulating inflammatory and tumor-associated molecular networks relevant to OC progression.

Nevertheless, the observed alterations in IL-17, NF-κB, MDK, and p21 levels remain correlative and do not establish direct causal relationships between these pathways and the cellular effects reported. Because pathway-specific inhibition and upstream or downstream functional analyses were not performed, the precise molecular mechanisms underlying these alterations cannot be definitively determined. Further targeted investigations will be required to clarify whether these signaling pathways directly mediate the antiproliferative and antimigratory effects observed in the present study.

## 5. Limitations

This study has several limitations that should be acknowledged. First, the findings are derived from in vitro experiments using a single ovarian cancer cell line (SKOV3), which may not fully capture the biological heterogeneity of OC. In addition, SKOV3 cells possess a p53-null background and therefore represent only a specific molecular subtype of ovarian cancer, which may limit the generalizability of the findings across different OC histotypes and genetic contexts. Second, although key inflammatory and tumor-associated biomarkers were evaluated, detailed mechanistic analyses at the transcriptional and signaling levels were not performed, and upstream regulatory mechanisms were not comprehensively investigated. Third, the lack of in vivo validation limits the direct translational interpretation of these results. In addition, although combination treatments were evaluated, a formal quantitative drug interaction analysis (e.g., combination index or synergy modeling) was not performed, and therefore the nature of potential drug interactions cannot be definitively characterized. Furthermore, certain metformin concentrations employed in the current in vitro experiments exceed clinically achievable plasma levels. Consequently, the translational implications of these findings should be interpreted with caution. Future studies utilizing pharmacologically relevant concentrations and more physiologically representative models are necessary to better evaluate their clinical applicability.

## 6. Future Research

Future studies should validate these findings in additional OC cell lines and in vivo models to better reflect tumor heterogeneity and microenvironmental interactions. The use of models with different genetic backgrounds, such as p53-mutant or BRCA-associated OC cells, may help determine whether the observed effects are consistent across molecular subtypes. Further mechanistic investigations focusing on the IL-17–NF-κB–MDK signaling axis and related upstream regulatory pathways are warranted. In addition, evaluating metformin-based combination strategies in preclinical models will be important to assess their therapeutic efficacy, toxicity profile, and pharmacodynamic interactions. The present study did not directly investigate upstream signaling pathways such as the AMPK/mTOR axis, which are known targets of metformin and resveratrol. Future studies examining AMPK activation and apoptosis-related markers may therefore provide deeper mechanistic insight into the molecular mechanisms underlying the observed cellular and biomarker changes.

## 7. Conclusions

In conclusion, this study demonstrates that boric acid and resveratrol enhance the antineoplastic effects of metformin in SKOV3 OC cells by suppressing cell proliferation and migration and modulating inflammatory and tumor-associated biomarkers, including IL-17, NF-κB, and MDK. The concomitant upregulation of p21 further indicates the involvement of cell cycle regulatory mechanisms in these responses. Together, these findings suggest that metformin-based combination strategies may represent a promising approach for targeting both proliferative and inflammatory pathways involved in OC progression. However, toxicity or safety assessments in non-cancerous cells were not performed in the present study; therefore, the safety profile of these treatments remains to be determined and requires further investigation in non-cancerous cellular models. In addition, NF-κB activity is primarily regulated through nuclear translocation rather than total protein abundance. As the present study assessed NF-κB levels using ELISA-based measurements in cell lysates, direct evaluation of NF-κB activation was not performed. Future studies employing complementary approaches, such as Western blotting or nuclear localization analyses, will be necessary to more precisely characterize NF-κB signaling activity.

## Figures and Tables

**Table 1:** Summary of treatment conditions applied to SKOV3 ovarian cancer cells.

Group	Metformin	Boric Acid	Resveratrol	Treatment Type
Control	–	–	–	Untreated control
Met-10	10 mM	–	–	Metformin monotherapy
Met-20	20 mM	–	–	Metformin monotherapy
Met-40	40 mM	–	–	Metformin monotherapy
Met-50	50 mM	–	–	Metformin monotherapy
BA-10	–	10 µM	–	Boric acid monotherapy
BA-20	–	20 µM	–	Boric acid monotherapy
BA-50	–	50 µM	–	Boric acid monotherapy
BA-100	–	100 µM	–	Boric acid monotherapy
Resv-25	–	–	25 µM	Resveratrol monotherapy
Resv-50	–	–	50 µM	Resveratrol monotherapy
Resv-100	–	–	100 µM	Resveratrol monotherapy
Met10 + BA100	10 mM	100 µM	–	Combination
Met10 + Resv25	10 mM	–	25 µM	Combination
Met40 + BA100	40 mM	100 µM	–	Combination
Met40 + Resv25	40 mM	–	25 µM	Combination

SKOV3 cells were treated with metformin, boric acid (BA), and resveratrol either as monotherapies or in combination.

**Table 2:** Comparison of quantitative migration inhibition percentages of SKOV3 cells.

	24 h	48 h	72 h	*p* Value
**100 µM BA**	48.83 [40.22–53.13]	28.10 [4.99–46.38]	0.0 [0.0–0.0] ^a^	**0.0371**
**25 µM Resv.**	20.37 [12.40–21.63]	42.16 [13.93–78.70]	0.0 [0.0–0.0]	0.1220
**10 mM Met.**	78.63 [69.33–85.72]	38.17 [31.26–95.08]	73.66 [63.90–95.40]	0.4211
**40 mM Met.**	55.84 [41.57–78.46]	50.16 [25.86–94.50]	-	>0.999
**10 mM Met. + 100 µM BA**	36.54 [31.81–57.91]	1.27 [8.45–27.16] ^b^	58.02 [49.57–58.26] ^c^	**0.0066**
**10 mM Met. + 25 µM Resv.**	45.36 [18.02–103.7]	25.02 [13.44–61.15]	29.20 [26.55–64.39]	0.6323
**40 mM Met. + 100 µM BA**	77.43 [59.52–92.50]	47.14 [25.77–52.76] ^d^	92.71 [76.27–95.20] *^,^**	**0.0280**
**40 mM Met. + 25 µM Resv.**	45.11 [33.56–51.40]	23.06 [14.99–47.40]	67.85 [67.48–71.92] ^e^	**0.0167**
***p* value**	0.0509	0.3679	**0.0057**	

All values obtained from three independent experiments are presented as median [minimum–maximum]. * *p* = 0.0414 vs. 100 µM BA group; ** *p* = 0.0414 vs. 25 µM Resv. group. ^a^ *p* = 0.0444 vs. corresponding 24 h; ^b^ *p* = 0.0180 vs. corresponding 24 h; ^c^ *p* = 0.0086 vs. corresponding 48 h; ^d^ *p* = 0.0378 vs. corresponding 72 h; ^e^ *p* = 0.0204 vs. corresponding 48 h.

**Table 3:** Comparison of findings of p21 immunoreactivities on SKOV3 cells.

p21	24 h	48 h	72 h	*p* Value
**Control**	8.82 [3.23–11.43]	8.11 [7.69–12.77]	13.26 [11.36–19.23]	0.0594
**100 µm BA**	18.75 [10.53–28.13]	20.69 [11.11–29.63]	16.68 [16.22–17.14]	0.7753
**25 µM Resv.**	20.00 [3.02–25.09]	31.82 [17.86–37.50]	35.14 [25.00–37.84]	0.0851
**10 mM Met.**	26.54 [24.13–33.33]	41.67 [36.36–50.00] ^b^	68.18 [66.67–72.22] ^d,i^	**<0.0001**
**10 mM Met. + 100 µm BA**	39.13 [24.14–50.00] ^a^	39.13 [29.17–53.57] ^c^	68.75 [52.63–72.22] ^ii^	**0.0073**
**10 mM MET + 25 µM Resv.**	28.85 [14.29–37.93]	34.78 [25.00–47.62]	70.43 [55.56–87.50] ^e,iii^	**0.005**
***p* value**	**0.0139**	**0.0007**	**0.0047**	

All values obtained from three independent experiments are presented as median and range. The percentage of p21-positive cells was quantified based on staining intensity and distribution across the experimental groups. ^a^ *p* = 0.0147 vs. control group; ^b^ *p* = 0.0016 vs. control group; ^c^ *p* = 0.01100 vs. control group; ^d^ *p* = 0.0417 vs. control group; ^e^ *p* = 0.0372 vs. control group; ^i^ *p* = 0.0021 vs. 24 h; ^ii^ *p* = 0.0193 vs. 24 h; ^iii^ *p* = 0.0194 vs. 24 h.

## Data Availability

The original contributions presented in this study are included in the article. Further inquiries can be directed to the corresponding author.
